# Readiness levels of intern nursing students during the transition to professional practice within the Al Jouf Region in Saudi Arabia

**DOI:** 10.1186/s12912-024-02106-5

**Published:** 2024-06-21

**Authors:** Abeer N. Alruwaili, Majed M. Alruwaili, Normajean Colby

**Affiliations:** 1 Nursing Administration & Education Department, College of Nursing, Jouf University, Sakaka, 72388 Saudi Arabia; 2 School of Nursing, Normajean Colby, Widener University, Chester, PA USA

**Keywords:** Readiness, Nursing students, Internship, Clinical practice, Saudi Arabia

## Abstract

**Background:**

The transition of newly graduated nurses into the workforce is recognized as a complex undertaking and has been examined extensively in the literature.

**Objective:**

This study aimed to assess the readiness levels of intern nursing students and investigate the factors affecting their transition to professional practice within the Al Jouf region in Saudi Arabia.

**Methods:**

The study employed a combination of descriptive, correlational, and qualitative methodologies to conduct its investigation. Data were acquired via an online questionnaire that included demographic information, the Nursing Practice Readiness Scale (NPRS), and two open-ended questions. A total of 135 nursing intern students were recruited to participate in the study. Benner’s “novice-to-expert” theory of clinical competence was utilized to guide the theoretical underpinning of the study.

**Results:**

Findings revealed that most intern nursing students (63.7%) exhibited a moderate level of readiness. Furthermore, 70.4% and 55.6% of the students showed moderate readiness in terms of their professional attitudes and patient-centeredness, respectively. More than one-third of the students demonstrated a high level of readiness in the self-regulation domain (36.3%), while a similar proportion indicated a high level of readiness in the domain of collaborative interpersonal relationships (33.3%). The students underscored their reliance on the education system as pivotal in enhancing their preparedness for clinical practice.

**Conclusion:**

Nursing internship programs contribute to a more comprehensive readiness of nurses for active participation in clinical practice as compared to traditional educational programs.

## Introduction

The readiness of new graduate nurses to enter the nursing profession has been debated since the transition from apprenticeship training programs to higher educational institutions [1]. In the clinical setting, nurses fulfill various roles, such as caregivers, communicators, educators, and advocates. New nurses need to be capable of meeting professional nursing requirements when assigned to a clinical site [2]. More importantly, they must possess multidimensional capabilities and characteristics to deliver safe, high-quality nursing care [3].

Work readiness is a multifactorial concept that warrants further exploration to gain a deeper understanding of its attributes, dimensions, and its significance in predicting a seamless and successful transition to professional practice [4]. Initially described by Caballero and Walker [5], work readiness has emerged as a construct predictive of favorable graduate outcomes, including successful integration into the workforce and sustained employment within the profession beyond the initial year [6]. It pertains to the degree to which new graduates exhibit qualities and competencies linked with success in the workplace [7].

The notion of readiness for practice pertains to the capacity of a graduate nurse to undertake the roles encompassing care provision, care design, management, coordination, and engagement as a member of the nursing profession [8]. However, the level of clinical preparedness is contingent upon the extent to which student nurses have been exposed to the various facets of nursing throughout their educational preparation program [9]. The assessment of practice readiness has consistently posed a challenge, given the increasing prominence of nursing student preparedness as a focal point for educational programs [9].

Transitioning new graduate nurses into the workforce is a challenging process and has been extensively studied in the literature. The initial nursing experience of new graduates typically occurs in a clinical setting, and their experiences and performance indicate that educational preparation is just as influential as the workplace environment in facilitating this transition [10]. Besides, perceived readiness is an important factor in the clinical adaptation of new graduate nurses, as it affects their confidence. New nurses who perceive themselves as lacking preparation for nursing practice struggle to apply what they have learned [11]. The successful transition of new nurses into practice is crucial for their long-term employment [12].

Furthermore, newly graduated nurses often encounter challenging situations. The difficulties include heavy responsibilities, work overload, fear of making errors, lack of confidence in their skills, unsupportive preceptors, end-of-life cases, and working in emergency departments [13]. To facilitate the transition from academic study to clinical practice, nursing students can benefit from a practice-based learning environment [14]. Providing assistance and ongoing education to graduate nurses, with a focus on improving their clinical experience, can promote a smoother transition and ensure safe nursing practice [15]. Internship programs, which is the case in Saudi Arabia, also serve as a form of experiential learning, allowing students to apply their classroom knowledge and theory to real-world situations and develop practical skills in a professional setting. This initiative provides valuable applied experience and helps students establish connections in their desired career paths [16].

According to a study conducted in Saudi Arabia by Alharbi et al. [17], the challenges faced by newly graduated nurses included difficulty in dealing with the health system and devices, fear of handling new patients, and difficulty in applying policies and procedures in the workplace. Also, Almotairy et al. [18] discovered that work readiness among newly graduated nurses in Saudi Arabia is relatively high. Work readiness was found to vary among students based on factors such as their country and university background, engagement in a second job, preferences for training hospitals, and selecting nursing as their primary field of study.

The healthcare system of Saudi Arabia is undergoing swift transformation to effectively address the evolving healthcare requirements of its populace. Within this context, nursing education in the nation has experienced noteworthy transformations, notably the incorporation of a one-year internship program serving as the concluding phase of nursing undergraduate education, which precedes the licensure examination. The internship program has been identified as a valuable mechanism for equipping nurses with aptitude and competence in preparation for the dynamic clinical milieu [19].

With the anticipated increase in the number of newly graduated nurses, the need for seamless transitioning into practice becomes paramount. As newly graduated nurses constitute the primary workforce for meeting the projected demand for additional nurses in Saudi Arabia, there is a pressing need for a deeper understanding of factors that facilitate their transition into practice and eventual success in the workplace [20]. While it has been hypothesized that the characteristics and attributes of work readiness play a crucial role in preparing new graduates for the workplace and predicting their workplace potential, there remains a significant gap in knowledge regarding work readiness among newly graduated nurses in Saudi Arabia. Moreover, it remains unclear which specific factors are significantly associated with work readiness in this context [18].

The current study aimed to assess the readiness levels of nursing intern students as they navigate the transition to clinical practice within the Al Jouf Region of Saudi Arabia. Additionally, the study sought to explore distinctive nursing intern attributes that could either facilitate or impede the seamless transition into clinical environments.

## Theoretical background

The theoretical framework underpinning the present study draws from Benner’s nursing theory, which delineates the progression of nurses’ competencies across five distinct stages. These stages encompass the “novice” category comprising individuals devoid of professional experience, followed by the “advanced beginner” level encompassing those who commence their roles as licensed registered nurses within clinical contexts. Subsequent stages encompass the “competent,” “proficient,” and the pinnacle stage of “expert” nurses [21]. . The presence of the mandatory one-year internship program in nursing education in Saudi Arabia is put the new graduate nurses from being “*novice*” toward the next level of “*advanced beginners*”, according to Benner’s theory. Thus; Benner’s theory was selected to be appropriate to guide the current study. It addresses the transitional phase nursing interns experience during the internship program, which helps new graduate nurses to be well-prepared for clinical practice [19]. Other nursing education theories can be of potential choice for this study, such as Henderson’s nursing needs theory, Mercer’s theory of maternal role attainment for nurses, and King’s theory of goal attainment [21]. The aforementioned three theories discuses the stages of nursing students experience to become professional nurses, but Benner’s theory of novice to expert still fits to be more suitable to the current study target population and variables being investigated [21]. As per Benner’s, nurses at the “novice” stage acquires the foundational knowledge and had no clinical experience and mal lacks the confidence to perform clinical tasks which is the situation of newly graduate nurses who moves to become “advanced beginners” within 2–3 years. The internship program in Saudi Arabia constitutes the transitional phase between the two described stages [19, 21].

## Methods

### Setting and sampling

This study employed a mixed-methods approach, incorporating both descriptive design and qualitative methods. The researchers adopted a convenience sampling strategy to enlist nursing intern students as participants. Inclusion criteria encompassed Saudi nursing intern students, irrespective of gender, who had successfully completed a Bachelor of Science in Nursing (BSN) program and had fulfilled a minimum of three months in their internship program within the Al Jouf region. Conversely, nursing interns who had concluded their intern program and obtained their graduation status, or those who were no longer situated within the Al Jouf region, were excluded from the study. To determine the minimum required sample size, a power analysis procedure was conducted with a 5% α error (95% significance level) and a 20% β error (80% power of the study). The estimated sample size was 103 nursing intern students. A total of 176 intern nursing students were invited to fill out the survey. In total, a sample of 135 intern nursing students responded to the invitation, representing approximately a 76% response rate. Consequently, the total sample size was ascertained to be 135 nursing intern students.

### Instruments

Data collection consisted of three sections: intern students’ demographics, the Nursing Practice Readiness Scale, and qualitative questions. The demographics section consisted of data pertaining to age, gender, grade point average (GPA), marital status, the specific unit of internship practice, presence of children, and prior experience/practice of nursing skills in a simulation lab were compiled. The second section was dedicated to the implementation of the 35-item Nursing Practice Readiness Scale (NPRS), designed for new graduate nurses and formulated by Kim and Shin [11]. This scale delineated five distinct factors: clinical judgment and nursing performance (16 items, Cronbach’s α = 0.83), professional attitudes (8 items, Cronbach’s α = 0.83), patient-centeredness (5 items, Cronbach’s α = 0.85), self-regulation (3 items, Cronbach’s α = 0.85), and collaborative interpersonal relationships (3 items, Cronbach’s α = 0.80). Participants responded to the scale’s items employing a Likert scale with four response points: strongly disagree (1), disagree (2), agree (3), and strongly agree (4). The scale’s possible range of scores was 35 to 140, with higher scores reflecting enhanced readiness. Notably, readiness levels were categorized as high (scores 112–140), moderate (scores 84–111), and low (scores 35–83). An assessment of internal consistency, gauged through the aggregation of all the elements within the scale, yielded a Cronbach’s α coefficient of 0.90.

The third section contained the two open-ended qualitative questions generated by researchers that were administered at the end of the quantitative survey, which were as follows:

1. What are the barriers and facilitators related to the readiness levels of nursing intern students?
2. What areas in nursing education require enhancement to ensure an improved and seamless transition process?

The research encompassed the inclusion of open-ended questions, designed to explore the participants’ subjective viewpoints regarding their readiness for practice. This methodological approach enabled participants to furnish responses that expanded upon diverse facets linked to the quantitative instrument employed. In the analysis of the qualitative data, the researchers adhered to a sequential six-step process delineated by Creswell (2017).

In applying Creswell’s six-step process for qualitative data analysis, researchers meticulously examined open-ended responses from participants to understand their subjective viewpoints on readiness for practice. Initially, they immersed themselves in the data to become familiar with its content and context. Subsequently, they generated initial codes to identify key concepts, followed by the identification of recurring patterns and categories. Through iterative review and refinement, the categories were validated and clearly defined, ensuring they accurately represented the data. Finally, the qualitative findings were integrated into the research report, complementing the quantitative data and offering a comprehensive understanding of participants’ perspectives. This systematic approach ensured rigor and reliability in analyzing the subjective aspects of readiness for practice.

The outcomes of the qualitative inquiries are succinctly presented in Table 6, outlining prominent categories that emerged from the meticulous analysis of the open-ended responses.

### Piloting

A pilot study was conducted on a group of 14 students before data collection to assess the feasibility, duration, and cost of a full-scale research project. No modifications were made, so the participants in the pilot study were included in this study. To ensure effective data collection, an online questionnaire was developed using the Qualtrics platform. This digital questionnaire was chosen for its convenience, ease of distribution, and efficient data management capabilities. The distribution of the questionnaire was carried out through official email channels as well as popular social media platforms such as WhatsApp and Telegram. By utilizing these diverse communication channels, the research team aimed to reach a wide range of participants and maximize the response rate. Participants were provided with a clear timeframe of approximately three weeks to complete the questionnaire, ensuring a reasonable and defined window for data collection.

The data collection phase spanned an extensive duration of three months, commencing on February 8 and concluding on April 10, 2023. This deliberate extension of the timeline was strategically orchestrated to facilitate substantial participation and ensure the accumulation of an ample number of responses, thereby engendering statistically meaningful outcomes. Throughout the data collection process, the research team remained proactive in monitoring responses, addressing any inquiries raised by participants, and ensuring the integrity and accuracy of the collected data.

### Ethical considerations

This study received ethical approval from the ethical committee affiliated with Jouf University. The research adhered diligently to the ethics guidelines for nursing research, encompassing the following considerations. The initiation of the data collection process ensued solely after the acquisition of the requisite approval from the Local Committee of Bioethics at Jouf University. This committee rigorously evaluated the well-being and rights of the participants, ensuring the ethical integrity of the study. In relation to the minimal potential for risk or harm, participation in the study was anticipated to carry no inherent risks. Participants were explicitly apprised that their responses would exert no influence whatsoever on their ongoing internship, evaluation procedures, or academic attainments.

Upon the commencement of the self-administered questionnaires, participants were meticulously furnished with a comprehensive overview of the study’s aims, significance, eligibility criteria, participants’ rights, and the prospective risks and benefits associated with their involvement. Furthermore, participants were unequivocally informed of their voluntary status within the study and were assured of their unreserved right to withdraw at any juncture without incurring any negative repercussions. As the questionnaire adopted a self-administered format, the formal process of obtaining written informed consent was not pursued. Instead, the return of the completed questionnaire was construed as indicative of implicit consent, adhering to the principles outlined by Polit and Beck [22].

### Statistical analyses

Data were sorted and classified, and the results are shown in tables below. The Statistical Package for the Social Sciences (SPSS Inc; version 27; IBM Corp., Armonk, NY, USA) was used to analyze the data. Descriptive statistics were used to delineate participants’ demographic details. For categorical variables, the data was conveyed through frequencies and percentages, while continuous variables were presented as mean values accompanied by standard deviations. The chi-square test was used to assess the relationship between the readiness level and intern nursing students’ characteristics. Ordinal logistic regression was also used to analyze the relationship between one or more independent variables and an ordinal dependent variable. The linear regression model analyzed the relationship between readiness level as the dependent variable and age, marital status coded as single 1 and married 0, having children coded as yes 1 and no 0, and clinical simulation training coded as yes 1 and no 0 as predictor variables. Statistical significance was set at *p* < 0.05.

## Results

Based on the characteristics of the intern nursing students in the study, the average age was found to be 24.14 years (SD = 3.17). The largest proportion of participants was clustered within the 23–27 age group, accounting for 57.8% of the total sample. Among the participants, female participants constituted a significant majority, encompassing 125 individuals, or 92.6% of the overall sample. When assessing marital status, a substantial proportion of participants were identified as single, accounting for 84.4% of the total, while a comparatively smaller segment reported being married (14.2%). Regarding academic performance, most participants fell within the GPA range of 3.76–4.5, constituting 44.4% of the participants, closely followed by those who held a GPA exceeding 4.5 (30.4%). Approximately one-third of the participants had been interns for a duration of 6–8 months. Of the participants, 90 reported receiving clinical training in a simulation lab, accounting for 66.7% of the total sample. Furthermore, more than half of the participants spent up to 4 h (57.8%) in the simulation lab, with 23.3% spending 5–10 h (see Table 1).

**Table 1:** Distribution of nursing student interns according to their characteristics (*N* = 135)

Items	*N*	%
Age		
18–22	44	32.6
23–27	78	57.8
28–32	9	6.7
> 32	4	2.9
Gender		
Male	10	7.4
Female	125	92.6
Marital Status		
Single	114	84.4
Married	19	14.2
Divorced	1	0.7
Widowed	1	0.7
Having Children		
Yes	17	12.6
No	118	87.4
GPA		
2–2.75	5	3.7
2.76–3.75	29	21.5
3.76–4.5	60	44.4
> 4.5	41	30.4
Internship Duration		
3 – <6 months	45	33.3
6 – <9 months	48	35.6
9–12 months	42	31.1
Clinical Training in a Simulation Lab		
Yes	90	66.7
No	45	33.3
If Yes, Time Spent (*N* = 90)		
Up to 4 h	52	57.8
5 to 10	21	23.3
11 to 19	12	13.3
> 20 h	5	5.6

The results of the NPRS confirms the phase of being in the transition between “novice” nurse in preparation to the next level of “advance beginner” as described by Benner’s theory [21]. In terms of clinical judgment and nursing performance, most intern nursing students (63.7%) exhibited a moderate level of readiness. Furthermore, 70.4% and 55.6% of the students showed moderate readiness in terms of their professional attitudes and patient-centeredness, respectively. Additionally, more than one-third of the students demonstrated a high level of readiness in the self-regulation domain (36.3%), while a similar proportion had a high level of readiness in the domain of collaborative interpersonal relationships (33.3%). Overall, 30.4% of the students achieved a high level of readiness according to the total readiness scale, while 60.7% had a moderate readiness level. Only 8.9% of the students had a low readiness level (Table 2). Being at the moderate level of readiness for the majority of interns is expected as Benner’s described that it would take a new graduate nurse about two to three years of clinical experience to achieve the “advance beginner” level of clinical competence [21].

**Table 2:** Distribution of nursing student interns according to their readiness levels (*N* = 135)

Items	High		Moderate		Low		M (SD)
	*N*	%	*N*	%	*N*	%	
Clinical judgment and nursing performance	37	27.4	86	63.7	12	8.9	47.9 (8.6)
Professional attitudes	13	9.6	95	70.4	27	20	24.3 (4.2)
Patient-centeredness	45	33.3	75	55.6	15	11.1	15.6 (2.9)
Self-regulation	49	36.3	61	45.2	25	18.5	9.3 (1.2)
Collaborative interpersonal relationship	45	33.3	77	57.1	13	9.6	9.86 (1.5)
Total	41	30.4	82	60.7	12	8.9	106.5 (17.7)

M, Mean, SD, standard deviation

Table 3 presents the findings related to the relationship between various factors and the readiness level of intern nursing students. The results indicate several significant associations. First, there was a statistically significant but slight correlation between the age of the intern nursing students and their readiness level, with a p value of < 0.05. Additionally, having children was also found to be related to readiness level, with a p value of < 0.05. Furthermore, a high correlation was observed between clinical training in a simulation lab and the readiness level of intern nursing students, with a p value of < 0.01. Moreover, the amount of time spent in training showed a slight but significant correlation with readiness level, with a p value of < 0.05. On the other hand, no significant relationship was found between gender, GPA, and the duration of being an intern with the domains of the readiness level of intern nursing students, with p values > 0.05.

**Table 3:** Relationship between readiness level and intern nursing students’ characteristics

Variable	Low *N* = 12		Moderate *N* = 82		High *N* = 41		Chi-square	*P*-value
	*N*	%	*N*	%	*N*	%		
Age							17.252	0.028
18–22	5	41.7	22	26.8	17	41.5		
23–27	4	33.3	50	61	24	58.		
28–32	1	8.3	8	9.8	0	0		
> 32	2	16.7	2	2.4	0	0		
Gender							2.867	0.580
Male	2	16.	6	7.3	2	4.9		
Female	10	83.3	76	92.7	39	95.1		
Marital Status							19.663	0.003
Divorced	0	0	1	1.2	0	0		
Married	3	25	15	18.3	1	2.5		
Single	8	66.7	66	80.5	40	97.5		
Widowed	1	8.3	0	0	0	0		
Having Children							6.742	0.034
Yes	3	25	13	15.8	1	2.5		
No	9	75	69	84.2	40	97.5		
GPA							3.882	0.180
2–2.75	1	8.4	3	3.7	1	2.5		
2.76–3.75	3	25	23	28.1	3	7.3		
3.76–4.5	4	33.3	23	28.1	23	56.1		
> 4.5	4	33.3	23	28.1	14	34.1		
Internship Duration							3.036	0.111
3 – <6 months	8	66.6	25	28	12	29.2		
6 – <9 months	2	16.7	28	34.1	18	43.9		
9–12 months	2	16.7	29	35.4	11	26.9		
Clinical Training in a Simulation Lab							9.919	0.007
Yes	8	66.	47	57.3	35	85.4		
No	4	33.4	35	42.7	6	14.6		
If Yes, Time Spent (*N* = 90)							15.131	0.047
Up to 4 h	5	41.6	28	34.1	19	46.3		
5 to 10	0	0	11	13.4	10	24.5		
11 to 19	2	16.7	5	6.1	5	12.2		
> 20 h	1	8.4	3	3.7	1	2.4		
None	4	33.3	35	42.7	6	14.6		

Tables 4 and 5 show that the highly significant model was detected through f test 6.312, p value.003. This explains 43% of the variation in readiness levels detected through R^2^ 0.43. Additionally, age and having children had a slight negative effect on readiness level at *p* < 0.05*. Single students and having clinical training in the simulation lab had a high frequency positive effect on readiness levels at p value = 0.001.

**Table 4:** Linear regression model for readiness level (*n* = 135)

Variables	Unstandardized Coefficients	Standardized Coefficients		*P*-value
	B	B	T	
Constant	0.120	0.083	1.500	0.057
Age	− 0.199	0.112	2.054	0.020
Marital Status “Single”	0.280	0.212	4.910	0.001
Having Children “Yes”	− 0.276	0.235	3.990	0.013
Clinical Training in Simulation Lab “Yes”	0.264	0.201	4.871	0.001

a. Dependent Variable: Readiness levels

b. Predictors: (constant): age, marital status “single”, having children “yes”, clinical training in a simulation lab “Yes”

**Table 5:** Regression coefficients for readiness level (*n* = 135)

Model	*R* ^2^	F	*P*-value
Regression	0.43	6.312	0.003**

The respondents were asked two open-ended questions at the end of the survey. First, “What factors would facilitate or hinder this experience?” was asked. The responses regarding barriers and facilitators related to readiness levels were developed into categories through content analysis by two of the researchers, first independently and then through collaboration reaching agreement (Table 6). The most common barriers noted included stress and uncooperative nursing staff who did not interact with or take interest in the nurse interns. The most common factors that were believed to be necessary to facilitate the transition experience were more guidance, support, and availability from nursing faculty clinical instructors during the internship experience, clear directions and expectations, and support and cooperation from hospital staff.

**Table 6:** Barriers and facilitators related to readiness levels of nursing intern students

Barriers related to readiness levels of intern nursing students	Facilitators related to readiness levels of intern nursing students
• Emotional stress	• Guidance and support in clinical settings
• Limited communication and nursing collaboration	• Clear directions and expectations
• Limited practical experience	• Support and effective communication
• Feelings of self-doubt and a lack of confidence	• Comprehensive orientation programs
• Lack of time management	• Modeling of decision-making and critical thinking
	• Adjusting to the work environment

A second question, “What do we have to do better in nursing education for a better and smoother transition?”, was answered by the respondents, and content analysis as described above resulted in categories representing respondent suggestions (Table 7). Overwhelmingly, respondents indicated that increased simulation experiences and increased clinical time with instructors with a strong clinical background providing meaningful guidance were needed for a better transition to practice. Consistent with responses related to barriers and facilitators, respondents also suggested improved communication, provision of clear directions, and improved collaboration between the hospital unit and the nursing school, as well as between the hospital staff and the nurse interns.

**Table 7:** Recommendations for nursing education

Recommendations
• Increased simulation – focusing on practice and application
• Increased clinical time- with exposure to greater number of patients/diagnoses
• Instructors with strong clinical backgrounds – providing guidance during clinical
• Clear expectations
• Improved communication and interaction between student interns and staff

## Discussion

The findings of this study indicate that the readiness levels of nursing intern students vary across different domains. Notably, a significant proportion of intern nursing students (63.7%) displayed a moderate level of readiness in terms of clinical judgment and nursing performance. This suggests that while they possess some competence in these areas, there is room for improvement. This comes in consistent with the Benner’s theory description of the transition between the “novice” to the “advance beginner” level of clinical competence [21]. Moreover, these findings are consistent with Kumm et al. [23], who found senior students to have low scores in critical thinking, prioritization, clinical judgment, and management of multiple responsibilities at the beginning of the capstone clinical experience. Likewise, Salem [24] stated that most nursing students had high readiness for practice and nursing performance.

Regarding professional attitudes, more than two-thirds of the students demonstrated a moderate level of readiness. This finding implies that a substantial portion of the students have a positive mindset and understand the importance of professionalism in their nursing practice. In the domain of patient-centeredness, more than half of the students showed a moderate level of readiness. This indicates that they possess a reasonable understanding of the importance of prioritizing patient needs and preferences. These results are consistent with the study by Rusch et al. [25], which revealed that students scored highest in professional attributes but lowest in time management, prioritization, and management of multiple patients. Additionally, Dudley et al. [26] stated that most newly graduated nurses had high professional readiness.

Interestingly, a noteworthy proportion of the students demonstrated a high level of readiness in the self-regulation domain (36.3%). This suggests that they possess strong self-regulatory skills, such as time management and self-reflection, which are crucial for effective nursing practice. Similarly, in collaborative interpersonal relationships, 33.3% of the students exhibited a high level of readiness, indicating their ability to effectively communicate and work with others in a team-based setting. These results are compatible with the study by Deasy et al. [27], which found that 58% of the students stated that they had self-confidence for clinical practice skills and 35% for theoretical knowledge.

When considering the overall readiness scale, 30.4% of the students achieved a high level of readiness, demonstrating competence across multiple domains. However, the majority of students (60.7%) displayed a moderate readiness level, indicating the need for further development and support in various aspects of their nursing education and training. Notably, only a small percentage of students (8.9%) had a low readiness level. This indicates that most nursing intern students possess a certain level of readiness and are generally well prepared to engage in clinical practice. These results can be attributed to approximately two-thirds of intern students attending clinical training in the simulation lab and approximately three-quarters of them having a high GPA. These results are supported by Güner [28], who mentioned that more than half of the students felt highly prepared to start work (57.6%). However, this is inconsistent with the study by Cleary-Holdforth and Leufer [8], who revealed that students did not feel adequately prepared for their role. Romyn et al. [29] also found that graduates did not feel prepared for their new role.

The current study mentioned that increased age and having children decrease readiness levels, while single students and having clinical training in the simulation lab improve readiness levels among intern nursing students. From the researchers’ point of view, these results may be contributed to older students and students with children having competing responsibilities, such as family obligations or work commitments, which could detract from their ability to fully engage in their nursing education. Students who are parents may experience additional stressors related to childcare arrangements, financial concerns, and household responsibilities, all of which may impact their perceived readiness for clinical practice. Furthermore, clinical training in the simulation lab may enhance readiness among nursing students, considering factors such as hands-on experience, exposure to diverse clinical scenarios, and mentorship opportunities.

These results are supported by Doughty et al. [30], who found that a structured program provided the support needed to ease the transition to practice. Additionally, Cleary-Holdforth and Leufer [8] stated that clinical training had a positive effect on students’ readiness to practice, while Güner [28] reported that older and male nursing students had a high readiness level.

Regarding barriers for nursing student interns related to their readiness levels, the current results reported that limited practical experience, feelings of self-doubt and a lack of confidence, lack of time management, limited communication and interprofessional collaboration, and emotional stress were significant factors. These results may be attributed to a lack of practical experience which is a common challenge reported by nursing students, especially interns. It can hinder their ability to apply theoretical knowledge to real-life clinical situations. Nursing students often experience self-doubt and lack of confidence, particularly in the clinical setting where they are expected to make critical decisions and provide patient care, particularly in situations in which they have had little to no experience. Also, effective time management is crucial for nursing students to balance academic demands, clinical responsibilities, and personal life. According to Wong et al. [31], transitional stress and displeasure with the work environment led to increased rates of new graduate nurses leaving their jobs. Moreover, Woods et al. [32] found that a lack of self-confidence impaired the readiness of nursing students. Furthermore, Adejumo et al. [33] detected that poor funding, lack of trained personnel, and social/environmental factors could affect nursing students’ readiness.

Facilitators for intern nursing students related to their readiness levels were provision of guidance and support in clinical settings, clear directions and expectations, facilitation of support and effective communication, comprehensive orientation programs, modeling of decision-making and critical thinking, and adjusting to the work environment. Such facilitators, attributed to open communication and support systems, may foster a conducive learning environment where students feel valued and encouraged to ask questions and seek clarification. The orientation education programs likely provide essential information, skills training, and familiarization with policies and procedures, enabling students to transition smoothly into their clinical placements. Timmins [34] mentioned that it is essential to foster an environment that actively encourages open communication and questioning to promote and develop good interpersonal communication skills among student nurses. Furthermore, school support during the transition improves students’ readiness practice level Lanahan et al. [35]. In addition, Kirkman et al. [36] reported that providing a chance for interprofessional communication and team building improves nursing students’ practice level.

Although the clinical judgment and nursing performance domain revealed that greater than half the participants scored in the moderate range for readiness for practice, there exists room for improvement. As the findings also revealed that clinical simulation training for nurse interns was significantly correlated with higher readiness to practice scores, it is recommended that quality simulation experiences be incorporated into all nursing education programs. Exposure to a variety of patient care situations through simulation may improve confidence, clinical knowledge, and time management skills, while addressing common barriers noted in the qualitative data such as limited practice exposure and experience, self-doubt, and a lack of of confidence. Further, nurse intern recommendations offered in response to the second open-ended question included the categories increased simulation experiences and exposure to a greater number of patient diagnoses.

Another category regarding the recommendations to enhance their nursing education experience reported by the participants included improved communication and interactions between the student interns and the hospital staff. In response to the open-ended question about barriers and facilitators for readiness to practice, participants identified limited communication and nursing collaboration as barriers and support and effective communication as facilitators. Of note, approximately one-fifth (20%) of the sample scored low on the NPRS domain of professional attitudes. Addressing uncooperative staff and negative interactions between hospital staff and the student nurse interns may positively influence readiness to practice scores in the professional attitudes domain. It is recommended that interprofessional collaboration be emphasized, by providing opportunities for students to work alongside other healthcare professionals to foster mutual respect, understanding, and teamwork. Further, simulation scenarios could be designed with/and include interprofessional components.

Lastly, as reported facilitators to readiness to practice, as reported by respondents in the qualitative data, included both comprehensive orientation programs as well as facilitating positive adjustment to the work environment, it is recommended that comprehensive orientation programs with clear expectations be developed. These orientation programs could include organizational policies, procedures, and culture, as well as training in communication and teamwork skills. Further, throughout these programs nurse interns could be offered continued guidance and support as these were facilitators highlighted by the interns in their qualitative responses. Mentoring, and networking opportunities could be included as well to facilitate ongoing growth, professional development, and learning among nurse intern students.

### Limitations

The current study had a few limitations. First, the study sample exclusively encompassed interns from a single university/region. Using only intern data from one institution limits the generalization of the study findings. Second, the open-ended question on the questionnaire about ‘factors that facilitate or hinder the transition to practice experience’ was asked as one question, rather than two separate questions. This approach inadvertently led to confusion in several responses, with uncertainty regarding whether respondents’ comments were about enhancing or hindering factors affecting the transition of nurses to professional practice.

Also, another limitation is the reliance on self-reported data, which may be subject to social desirability bias and may not fully capture the nuances of readiness levels among nursing students. Additionally, the study’s focus on a specific region within Saudi Arabia may limit the generalizability of findings to other contexts.

To mitigate these limitations, future research to ensure a diverse sample, including students from various regions and educational institutions, could enhance the external validity of findings and provide a more representative picture of readiness levels among intern nursing students in Saudi Arabia. Furthermore, incorporating objective measures of readiness, such as clinical assessments or supervisor evaluations, alongside self-reported data, could help validate the findings and mitigate potential biases. Collaborating with multiple stakeholders, including nursing schools, healthcare institutions, and regulatory bodies, may also provide valuable insights and perspectives on the factors influencing the transition of nursing students into professional practice.

### Recommendations

Subsequent investigations could improve the understanding of the readiness levels of intern nursing students and the factors affecting the transition of nurses to professional practice. Future studies could broaden the scope of this research by including more universities or regions. Employing a qualitative methodology involving interviews or focus groups could enable a more comprehensive exploration of the barriers and facilitators to readiness. Moreover, the possibility of implementing the readiness scale at various points during the internship experience holds promise for yielding richer insights. Recommendations gleaned from this study’s findings include providing more opportunities for clinical simulation opportunities for student nurse interns both in school and during their internship, which also include interprofessional collaboration scenarios. Further, it is recommended to develop comprehensive orientation programs with clear directives for the student nurse interns regarding both organizational policy and procedures, as well as a structured clinical practice orientation to support and guide their learning.

## Conclusion

The current study has unveiled the variability in readiness levels among nursing intern students across diverse domains. Approximately one-third of the students attained a high level of readiness as indicated by the overall readiness scale, signifying proficiency in multiple facets. A considerable portion of students demonstrated a moderate readiness level, underscoring the necessity for further growth and support in various dimensions of their nursing education and training. However, only a minor fraction of students exhibited low readiness levels, signifying that most nursing intern students possess a certain level of readiness and are generally well-equipped to embrace clinical practice.

Furthermore, the study elucidated several obstacles impeding readiness levels in intern nursing students. The barriers included limited practical exposure, self-doubt, deficient self-confidence, deficient time management skills, constrained communication and interprofessional collaboration abilities, and emotional strain. These outcomes underscore the imperative of targeted interventions and support mechanisms to surmount these barriers and elevate students’ readiness levels. Recommendations include increased opportunities for simulation training to enhance confidence and clinical knowledge skills in diverse clinical scenarios; incorporating strategies to improve collaboration with the healthcare team, particularly communication between nursing staff and student nurse interns; and structured orientation programs. However, several facilitators were identified that can effectively bolster readiness levels among nursing intern students. These include furnishing guidance and support within clinical settings, rendering lucid directions and expectations, nurturing decision-making, and critical thinking proficiencies, aiding students in adapting to their work milieu, delivering comprehensive orientation programs, and fostering effective communication and teamwork. These facilitators can play a pivotal role in enhancing students’ preparedness for practice, equipping them to address the challenges inherent to their nursing careers effectively.

Overall, the findings from this study offer valuable insights into the readiness levels of nursing intern students and illuminate the factors shaping their aptitude for clinical practice. By addressing the identified barriers and capitalizing on the facilitators, nursing education programs and healthcare establishments can significantly enhance the readiness of intern nursing students. Ultimately, this holistic approach contributes to elevated patient care outcomes and the cultivation of capable and confident nursing professionals.

## Data Availability

The datasets generated and/or analyzed during the current study are not publicly available due funded ongoing project policy but are available from the corresponding author on reasonable request.
